# Heatmap centrality: A new measure to identify super-spreader nodes in scale-free networks

**DOI:** 10.1371/journal.pone.0235690

**Published:** 2020-07-07

**Authors:** Christina Durón

**Affiliations:** Department of Mathematics, University of Arizona, Tucson, Arizona, United States of America; Unviersity of Burgundy, FRANCE

## Abstract

The identification of potential super-spreader nodes within a network is a critical part of the study and analysis of real-world networks. Motivated by a new interpretation of the “shortest path” between two nodes, this paper explores the properties of the heatmap centrality by comparing the farness of a node with the average sum of farness of its adjacent nodes in order to identify influential nodes within the network. As many real-world networks are often claimed to be scale-free, numerical experiments based upon both simulated and real-world undirected and unweighted scale-free networks are used to illustrate the effectiveness of the proposed “shortest path” based measure with regards to its CPU run time and ranking of influential nodes.

## 1 Introduction

Networks provide a framework to model complex systems, utilizing nodes and edges to depict the interactions between system components. While various tools have been developed to analyze real-world systems, nodal centrality measures are one of the more prominently used network analysis techniques to quantify the influence of a node with respect to other nodes within the network [1]. Some of the more well-known centrality measures include the degree centrality (*C*_*D*_) [2], eigenvector centrality (*C*_*E*_) [3], closeness centrality (*C*_*C*_) [4], and betweenness centrality (*C*_*B*_) [5]. The utilization of centrality measures on networks to identify influential nodes can lead to a more comprehensive understanding of the dynamics and behavior of real-world systems, such as the identification of the most influential individuals in a social network, the key airports in a transportation network, or the super-spreaders in a disease [6]. Past applications of the four well-known centralities, along with various generalizations of the measures, on real-world networks include the Internet [7,8], transportation systems [9,10], biological systems [11–13], and social systems [14,15].

However, the identification of influential nodes through centrality measures depends upon the network structure. Generally, a node with a higher centrality value is considered more influential than the other nodes, where the value numerically quantifies the level of influence a node has with respect to the network’s topology. For instance, the degree centrality of a node is an indication of the number of neighbors connected to it. An extension of degree centrality, the eigenvector centrality of a node is a measure of the degree of both the node and its neighbors. The closeness centrality of a node quantifies the shortest path distances to every other node in the network, while the betweenness centrality of a node represents the fraction of shortest paths that go through the node among all shortest paths between every node pair in the network.

Yet, each centrality measure comes with its own limitation. For example, the degree centrality considers only local information about nodes, assigning a greater influence to nodes that are connected to a greater number of adjacent nodes [2]. The eigenvector centrality measures the influence of a node based upon the influence of its neighbors, and as a result, often identifies a set of influential nodes that all are located within the same region of the network [16]. The closeness centrality quantifies a node’s influence based upon its shortest path distances to the other nodes in a network, which, consequently, is why its numerical range is often small [17]. Finally, because the betweenness centrality utilizes the global information of the network by ranking nodes higher if they lie on many shortest paths within a network, as the size of the network grows, the computational complexity of the betweenness centrality increases [18].

In fact, with respect to the time complexity of the algorithms to compute the centrality measure for an individual node in the network, the betweenness centrality is the slowest among the four well-known measures. Consider an arbitrary, unweighted, and undirected network with |*V*| = *N* nodes and |*E*| = *M* edges. For the shortest path based measures, the calculation of the closeness and betweenness value of an individual node in the network requires an implementation of the breadth-first search (BFS) algorithm to determine the shortest path from one node to all other nodes, such that the closeness and betweenness centralities run in *O*(*N*+*M*) and *O*(*N*^2^+*NM*) time, respectively [6]. Since the betweenness value for an individual node requires the calculation of the fraction of shortest paths that go through the node among all shortest paths between every node pair in the network, the BFS algorithm must be executed on every node in the network which causes the betweenness measure to amass a larger computational overhead.

While a node with a very large degree is called a “hub” node [19–22], a node with immense infectivity in a real-world network is considered a “super-spreader” (i.e., a node that maximizes its impact on other nodes, such as in the case of facilitating the spread of information or the propagation of a disease) [22–24]. Previous studies have assumed that a hub node is equivalent to a super-spreader [22], and while it is true in some cases, there are many real-spreading processes that cannot be completely described by this process (e.g. a hub node may not contact all of its neighbors within one-time step in an epidemic contact network; additional scenarios are detailed elsewhere [22]). Therefore, although high degree nodes, high betweenness nodes, and high closeness nodes have been identified as good initial spreaders [22], the contribution of this paper is the proposal of the heatmap centrality, a measure which utilizes features from all three measures to strike a balance between accuracy and algorithmic simplicity in the identification of influential nodes. Motivated by a different interpretation of the “shortest path” between two nodes, this paper, in particular, aims to explore the properties of the proposed centrality as a potentially viable measure in the identification of super-spreaders (i.e., nodes that are influential in the flow of information) within real-world networks.

Define the farness of a node as the sum of the length of shortest paths from the node to all other nodes in the network. Then the heatmap centrality of a node is defined, simply, as the difference in the node’s farness and the average farness of its neighbors. The theoretical intuition behind the proposed measure is that a node is likely to lie on the shortest paths for several pairs of nodes within the network if the node’s farness is smaller than the average of its neighbors, resulting in a negative heatmap value. If a node and all of its neighbors have a similar farness, then information can flow through any of those nodes and neither of the nodes may be more influential than the others. For example, if an individual and each of its immediate neighbors are infected, then neither of them necessarily possess more infectivity nor influence than the other. Yet, if an individual is the only one infected among its immediate neighbors, then the individual possesses more infectivity and influence than the others. Using the above arguments, a node with a large negative heatmap centrality is more likely to control the diffusion of information among other nodes and possess a greater amount of infectivity.

As many real-world networks are often claimed to be scale-free [25] (e.g., social networks [26], the Internet [27], protein-protein interaction networks [28,29], and airline networks [30,31]), the properties of the heatmap centrality measures are explored in this paper through experimental studies involving both simulated and real-world scale-free networks. Generally, a network is considered “scale-free” if the fraction of nodes with degree *k* follows a power-law distribution *k*^−*γ*^, where *γ*>1 [25]. Yet, alternative versions of the “scale-free hypothesis” have included weaker requirements, such that the power law only needs to hold in the upper tail of the degree distribution [32], or stronger requirements, such as requiring 2<*γ*<3 [33,34]. As a consequence of the various interpretations of the “scale-free”, different researchers can apply the term to slightly different concepts, ultimately complicating efforts to evaluate the relationship and properties between the centrality measures on scale-free networks [25]. To avoid any confusion with the term “scale-free”, in this paper, a “scale-free” network is defined as a simple, undirected network whose degree distribution follows a power-law distribution *k*^−*γ*^, where *γ*>1. Additionally, any network that is either too dense or too sparse is not considered, as networks with such densities are not plausibly scale-free [25].

To evaluate the algorithmic performance and efficiency on simulated scale-free networks, the CPU (central processing unit) times and the Spearman-rank and Kendall-rank correlation coefficients of the proposed heatmap measure are calculated on networks of various size *N* and density *d*. To evaluate the algorithmic efficiency on real-world scale-free networks, three experiments are performed to compare the nodal ranking of the proposed measure with the rankings with respect to the degree, eigenvector, closeness, and betweenness centralities: A comparison of the top-10 ranked nodes, a comparison of both the Spearman-rank and Kendall-rank correlation coefficients between each pair of measures, and a comparison of the spreading capability of the top-10 nodes using a modification of the standard Susceptible-Infected (SI) model [35–38]. Based upon the results of the experiments performed on both the simulated and real-world scale-free networks, the heatmap centrality may be considered as a potentially viable measure in the identification of super-spreader nodes in scale-free networks.

The rest of the paper is organized as follows. Section 2 provides preliminary material, such as definitions and notations that will be referred to throughout the paper, and an overview of the four well-known centrality measures is provided. Section 3 introduces the proposed heatmap centrality measure, discusses its time complexity, and provides a toy example to highlight the properties of each centrality measure, as well as the advantages of the proposed measure. Section 4 details the two rank correlation coefficients, Spearman and Kendall, that are used to compare the rankings with respect to two centrality measures. An example calculation of each correlation with respect to the betweenness and heatmap centralities is also included in this section. In Section 5, an overview of the SI model is provided, while Section 6 describes the datasets and introduces the evaluation methodologies used to study the accuracy of each measure. The analysis of the experimental results is also included in the section. Section 7 concludes the paper and details future work.

## 2 Preliminary concepts of a graph

Formally, an undirected and unweighted network is defined as an abstract graph *G* = (*V*,*E*) where *V* is the finite, nonempty set of nodes, and *E* is the set of edges. Denote the set of edges *E* = {*e*_*j*,*k*_|*v*_*j*_,*v*_*k*_∈*V*} by the set of connections between nodes *v*_*j*_ and *v*_*k*_. Because *G* is undirected, if *e*_*j*,*k*_∈*E*, then *e*_*k*,*j*_∈*E*.

If *e*_*j*,*k*_∈*E*, then nodes *v*_*j*_ and *v*_*k*_ are considered adjacent, where node *v*_*j*_ is known as a neighbor of node *v*_*k*_, and node *v*_*k*_ is a neighbor of node *v*_*j*_. The neighborhood of node *v*_*j*_∈*V* is the set of the neighbors of *v*_*j*_∈*V*.

A path *P*(*v*_*j*_, *v*_*k*_) between nodes *v*_*j*_ and *v*_*k*_ is defined as a sequence of edges that connect adjacent nodes, and the length of a path *P*(*v*_*j*_, *v*_*k*_) is defined as the sum of the edges in *P*(*v*_*j*_, *v*_*k*_). Let ${P(v}_{j},v_{k})$ be the set of all paths between nodes *v*_*j*_ and *v*_*k*_. Then $P_{s}(v_{j},v_{k})\in P(v_{j},v_{k})$ is defined as a shortest path (i.e., a path of minimum length) between nodes *v*_*j*_ and *v*_*k*_ with length *s*(*v*_*j*_, *v*_*k*_).

Finally, define a graph as connected if there exists a path between every pair of its nodes. In this paper, only simple, undirected, unweighted, and connected networks are considered, where a simple graph, by definition, does not contain duplicate edges or loops (i.e., an edge that connects a node to itself).

### 2.1 Centrality measures

A centrality measure is a function C:V→R that assigns a value to each node within the network. This value numerically quantifies the influence of a node with respect to the network structure, where a node with a higher centrality value is usually considered more influential than the other nodes. Centrality measures do not consider node-level covariate information (i.e., node characteristics that “co-vary” with the network). Although many centrality measures have been proposed to rank nodes within a network according to their level of influence, the well-known measures for characterizing the centrality of a node within a network include the degree, eigenvector, closeness, and betweenness centralities.

Consider a simple, undirected, unweighted, and connected network *G* = (*V*,*E*) with *N* = |*V*| nodes and *M* = |*E*| edges. Then the network *G* can be described by an adjacency matrix *A* = {*a*_*i*,*j*_} of size *N*×*N*, where *a*_*i*,*j*_ = 1 if nodes *v*_*i*_ and *v*_*j*_ are connected by edge *e*_*i*,*j*_, and *a*_*i*,*j*_ = 0 if otherwise.

### 2.2 Degree centrality

The degree centrality for node *v*_*i*_, denoted as *C*_*D*_(*v*_*i*_), is defined [2] as

$${C_{D}(v}_{i})=\sum_{j=1}^{N}a_{i,j}$$

The degree centrality measures the influence of a node by the number of edges connected to it, where a node with a high degree value is a highly connected node within the network and thus, involved in a large number of interactions. The degree centrality is considered a local network measure as it does not take the structure of the rest of the network into account. The time complexity to compute the degree centrality of one node, *C*_*D*_(*v*_*i*_), is *O*(*N*) since there are *N* entries in the row corresponding to each node in the adjacency matrix *A* [6]. Consequently, the calculation of the degree centrality of all *N* nodes in the network requires *O*(*N*^2^) time in a dense network (i.e., *M* = *N*^2^), and *O*(*M*) time in a sparse network (i.e., *M*<*N*(*N*−1)/2).

### 2.3 Eigenvector centrality

Let *λ*_1_,*λ*_2_,…,*λ*_*N*_ denote the eigenvalues of the adjacency matrix *A* = {*a*_*i*,*j*_} of network *G*. Then the largest eigenvalue of matrix *A* is *λ*_max_ with corresponding eigenvector *e* = [*e*_1_,*e*_2_,…,*e*_*N*_]^*T*^ such that $\lambda_{\max}e_{i}=\sum_{j=1}^{N}a_{i,j}e_{j}$. Then, the eigenvector centrality for node *v*_*i*_, denoted as *C*_*E*_(*v*_*i*_), is defined [3] as

$${C_{E}(v}_{i})=\lambda_{\max}^{-1}\sum_{j=1}^{N}a_{i,j}e_{j}$$

Intuitively, the eigenvector centrality quantifies node *v*_*i*_ as influential if it is connected to other influential nodes within a network [17], thus calculating both the direct and indirect influence of the node. As a result, the eigenvector centrality is considered a measure of the global network connectivity. Since the power method is used in the calculation of the eigenvector centrality, there is no “proven” time complexity as the exact time complexity depends upon a number of factors, such as the speed of the convergence of the normalized eigenvector to *λ*_max_.

### 2.4 Closeness centrality

The closeness centrality for node *v*_*i*_, denoted as *C*_*C*_(*v*_*i*_), is defined [4] as

$${C_{C}(v}_{i})=\frac{1}{\sum_{j=1}^{N}s(v_{i},v_{j})}$$

or, more simply, as the reciprocal of farness, where farness is defined as the sum of the length of the shortest paths between node *v*_*i*_ and all other nodes in the network. Intuitively, the closeness centrality measures how quickly information can spread from node *v*_*i*_, utilizing the idea that a node is close to all nodes within the network and not just close to its neighbor. As a result, the closeness centrality is considered a measure of the global network connectivity. Furthermore, the time complexity needed to calculate the closeness centrality of one node, *C*_*C*_(*v*_*i*_), is *O*(*N*+*M*) since the centrality utilizes breadth-first search (BFS), an algorithm that runs in *O*(*N*+*M*) time, to determine the shortest path from one node to all other nodes [6]. Therefore, in order to compute the closeness centrality of all *N* nodes in the network, the BFS algorithm is implemented on each of the *N* nodes such that the time complexity for the closeness centrality is *O*(*N*(*N*+*M*)) = *O*(*N*^2^+*NM*). For all purposes, this is *O*(*NM*).

### 2.5 Betweenness centrality

The betweenness centrality for node *v*_*i*_, denoted as *C*_*B*_(*v*_*i*_), is defined [5] as

$${C_{B}(v}_{i})=\sum_{i\neq j\neq k}^{N}\frac{\sigma_{v_{j},v_{k}}(v_{i})}{\sigma_{v_{j},v_{k}}}$$

where $\sigma_{v_{j},v_{k}}$ is the number of shortest paths between nodes *v*_*j*_ and *v*_*k*_, and $\sigma_{v_{j},v_{k}}(v_{i})$ is the number of shortest paths between nodes *v*_*j*_ and *v*_*k*_ that pass through node *v*_*i*_. The interactions of two nonadjacent nodes depend upon other nodes, which generally lie on the shortest paths between the two nodes [39]. Thus, the betweenness centrality considers a node influential if it lies on a large fraction of shortest paths between a pair of nodes within the network. Intuitively, the betweenness centrality measures how much information is likely to flow through node *v*_*i*_. Since the betweenness centrality takes the structure of the entire network into account, it is considered a global network measure.

To calculate the betweenness centrality of a node within the network, the fastest known algorithm is Brandes [40] which performs two basic steps. First, an augmented BFS starts from node *v*_*j*_ and computes $\sigma_{v_{j},v_{k}}$ for every node *v*_*k*_. Secondly, during the accumulation phrase, the BFS constructed in the previous step is utilized to calculate the value of *C*_*B*_(*v*_*i*_) for every node *v*_*i*_. The time complexity of a BFS in the first step is *O*(*N*+*M*), while the time complexity in the accumulation phase is also *O*(*N*+*M*) as the maximal number of steps is bounded by both the number of parents, *O*(*M*), and the nodes accessed, *O*(*N*) [41]. Since this computation is performed for each node, then the calculation of the betweenness centrality on all *N* nodes using the Brandes algorithm requires a time complexity of *O*(*N*(*N*+*M*)) = *O*(*N*^2^+*NM*), which for all purposes, simplifies to *O*(*NM*).

Although the closeness and betweenness centralities each require *O*(*NM*) time to calculate the shortest paths for all *N* nodes in the network, the closeness centrality is a relatively less time-consuming measure in comparison, as it does not require much of the post-processing work (i.e., calculating the number of shortest paths that pass through the node) that is required for the betweenness centrality. In particular, for each node *v*_*i*_, the *N* shortest paths trees from the BFS algorithm must be traced through in order to determine the number of shortest paths that pass through *v*_*i*_ [6]. Therefore, calculating the betweenness centrality for one node, *C*_*B*_(*v*_*i*_), could take an additional *O*(*NM*) time such that the overall time complexity to compute the betweenness centrality for all the nodes in the network would be *O*(*N*^2^+2*NM*), or simply *O*(*NM*) [6].

## 3 Heatmap centrality

A new centrality measure, termed the heatmap centrality, utilizes both local and global network information by comparing the farness of each node (i.e., the global network information) with the average sum of the farness of its neighbor nodes (i.e., the local network information). In particular, the heatmap centrality for node *v*_*i*_, denoted as *C*_*HM*_(*v*_*i*_), is defined formally as

$${C_{HM}(v}_{i})=\sum_{j=1}^{N}s(v_{i},v_{j})-\frac{\sum_{j=1}^{N}a_{i,j}∙\sum_{k=1}^{N}s(v_{j},v_{k})}{\sum_{j=1}^{N}a_{i,j}}$$

The heatmap measure identifies the “hot spot” node within its neighborhood, as it considers a node with a smaller farness than that of the average of its neighbors to be an influential node within the network. Intuitively, a node with the smaller farness among that of its neighbors is more likely to have information pass specifically through it, rather than through any of the adjacent nodes. When the sign of *C*_*HM*_(*v*_*i*_) transitions from negative to positive, then the average sum of the farness of the neighbors of *v*_*i*_ becomes smaller than that of node *v*_*i*_, decreasing the likelihood of information passing specifically through node *v*_*i*_. Therefore, using this intuition, the heatmap centrality can be considered a “shortest path” based measure and utilized in the identification of super-spreader nodes that control the flow of information within a scale-free network.

**Fig 1:**
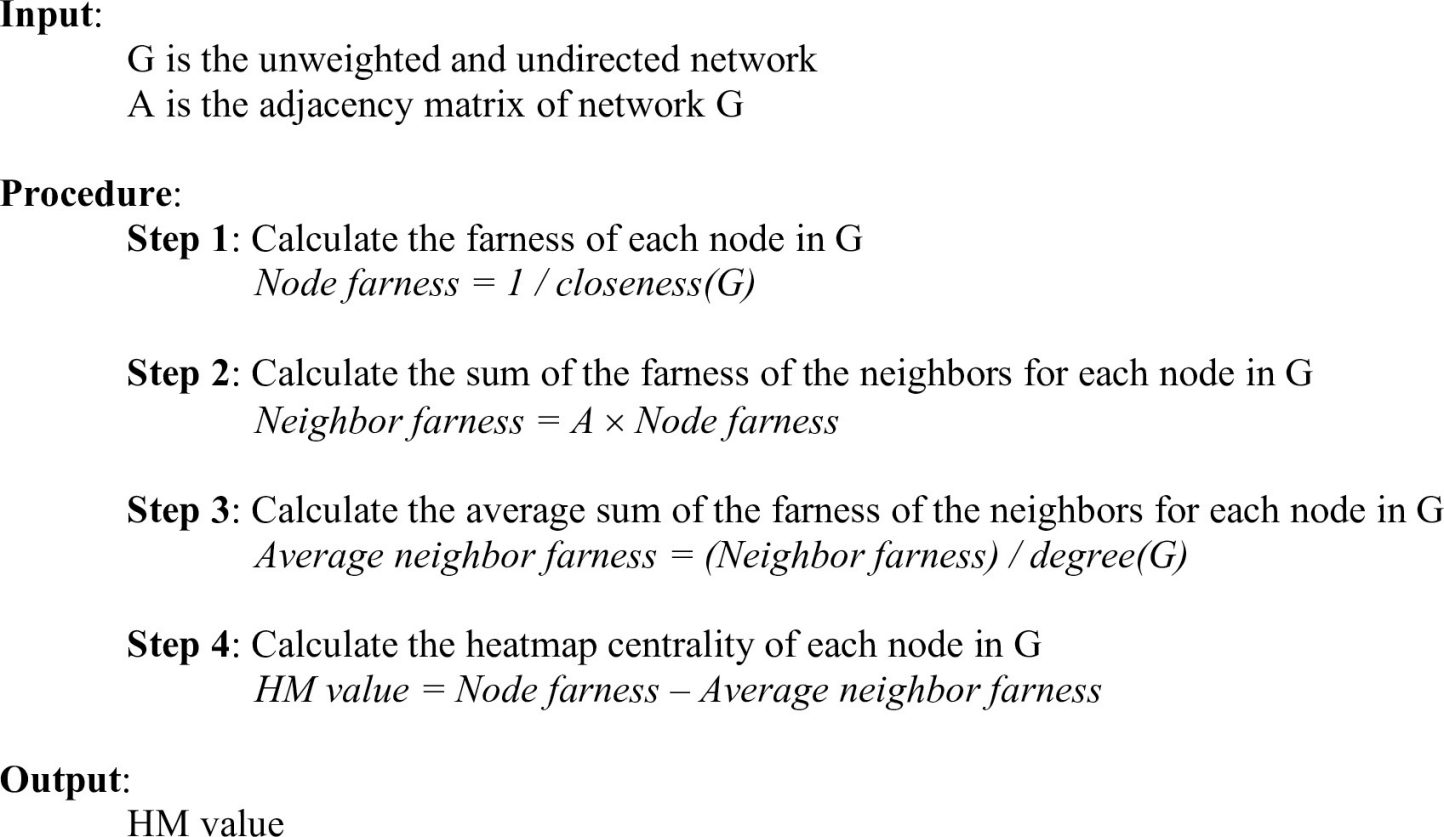
Pseudocode. The heatmap centrality algorithm pseudocode.

Consider the pseudocode for the heatmap centrality algorithm in Fig 1 and node *v*_*i*_ with, say, *k* neighbors. In Step 1, the calculation of the farness of node *v*_*i*_ and its *k* neighbors requires *O*((*k*+1)(*N*+*M*)) time as BFS needs to be executed (*k*+1)-times. In Step 2, the sum of the farness of the *k* neighbors requires *O*(*k*) time, while Step 3 requires *O*(*k*+1) time to calculate both the degree of node *v*_*i*_ and the average sum of the farness of its neighbors. Finally, the subtraction in Step 4 requires *O*(1) time. Therefore, the calculation of the heatmap centrality for an individual node, *C*_*HM*_(*v*_*i*_), with *k* neighbors requires *O*((*k*+1)(*N*+*M*)+2*k*+2) time, which for all purposes, simplifies to *O*(*N*+*M*).

Therefore, calculating the heatmap centrality for all *N* nodes within a sparse network requires a time complexity of *O*(*N*(*N*+*M*)+3*M*+2*N*) = *O*(*N*^2^+*NM*+3*M*+2*N*), which for all purposes, simplifies to *O*(*NM*). Note that Step 1 requires *O*(*N*(*N*+*M*)) time to run BFS for each node, Step 2 requires *O*(2*M*) time to sum the farness of the neighbors for each node, Step 3 requires *O*(*M*+*N*) time to compute both the degree of each node and the average sum of the farness of the neighbors for each node, and Step 4 requires *O*(*N*) time to perform the subtraction for each node. Consequently, for a dense network, the calculation of the heatmap centrality for all *N* nodes requires a time complexity of *O*(*N*(*N*+*M*)+2*N*^2^+2*N*) = *O*(3*N*^2^+*NM*+2*N*).

### 3.1 Toy example

**Fig 2:**
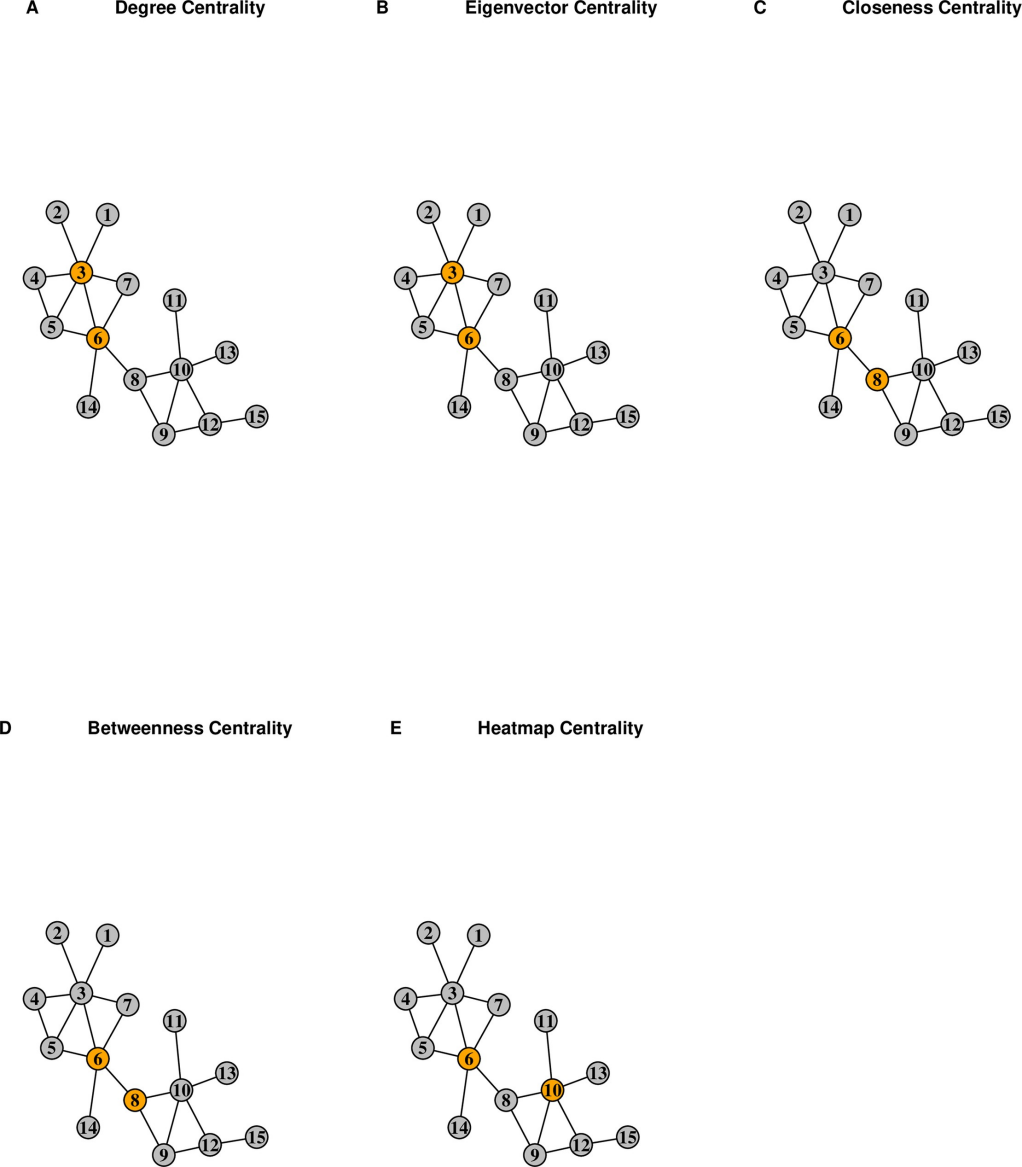
The rankings of nodes in the example network. The top two-ranked nodes in the example network *G* with 15 nodes and 19 edges with respect to the (A) degree, (B) eigenvector, (C) closeness, (D) betweenness, and (E) heatmap centrality measures are colored orange, while the remaining 13 nodes are colored grey.

Fig 2 displays a simple, undirected, unweighted, and connected network *G* with 15 nodes and 19 edges. The advantages of the proposed measure are detailed by comparing the rankings of the nodes in the network with respect to the degree centrality (*C*_*D*_), eigenvector centrality (*C*_*E*_), closeness centrality (*C*_*C*_), betweenness centrality (*C*_*B*_), and heatmap centrality (*C*_*HM*_). The centrality value of each node and its ranking with respect to the five measures are provided in Table 1. Unsurprisingly, node 3 is top-ranked by both the degree and eigenvector centralities, as it is the most connected node within the network. In addition, node 6 is top-ranked by the closeness, betweenness, and heatmap measures, since its close proximity to all the nodes within the network allows it to control the diffusion of information. Yet, although node 6 is second-ranked by both the degree and eigenvector measures, and node 8 by the closeness and betweenness measures, the heatmap centrality ranks node 10 second. Nodes 6 and 8 both lie on the path that bridge the two components of network *G*, such that information passing through node 6 must pass through node 8 in order to reach the nodes on the right side of the network. As a result of its positioning on the bridge path, node 8 is given large closeness and betweenness values strictly due to its adjacency to node 6. But the heatmap centrality does not assign a node a high rank necessarily due to a topological advantage. Instead, the proposed measure leverages the farness of a node’s neighbors to provide a ranking that is more reflective of how information flows throughout the network.

**Table 1:** The ranking of all 15 nodes in the example network *G* with respect to the degree (*C*_*D*_), eigenvector (*C*_*E*_), closeness (*C*_*C*_), betweenness (*C*_*B*_), and heatmap (*C*_*HM*_) centrality measures.

Node *v*_*i*_	*C*_*D*_(*v*_*i*_)	Rank	*C*_*E*_(*v*_*i*_)	Rank	*C*_*C*_(*v*_*i*_)	Rank	*C*_*B*_(*v*_*i*_)	Rank	*C*_*HM*_(*v*_*i*_)	Rank
**1**	1	10	0.2993	9	0.022	11	0	8	13	10
**2**	1	11	0.2993	10	0.022	12	0	9	13	11
**3**	6	1	1	1	0.030	3	31	4	-6.667	3
**4**	2	8	0.518	5	0.022	10	0	10	10.5	9
**5**	3	4	0.730	3	0.028	6	4.5	7	0.667	6
**6**	5	2	0.919	2	0.036	1	55.5	1	-7.2	1
**7**	2	9	0.575	4	0.027	7	0	11	6.5	8
**8**	3	5	0.491	6	0.034	2	48	2	-3	4
**9**	3	6	0.3343	8	0.029	5	9	6	0	5
**10**	5	3	0.388	7	0.030	4	34	3	-6.8	2
**11**	1	12	0.116	13	0.022	13	0	12	13	12
**12**	3	7	0.238	12	0.023	9	13	5	1.667	7
**13**	1	13	0.116	14	0.022	14	0	13	13	13
**14**	1	14	0.275	11	0.024	8	0	14	13	14
**15**	1	15	0.071	15	0.018	15	0	15	13	15

## 4 The rank correlation coefficients

The ranking of a node with respect to a centrality measure quantifies the node’s influential seating if the nodes in the network are ordered in the decreasing order of the centrality values. As there are many different centrality measures used to rank the nodes of a network by their level of influence, rank correlation coefficients may be utilized to determine the similarity of two centrality rankings. Intuitively, the correlation coefficient between two variables will be high when two variables have a similar rank, and low when two variables have a dissimilar rank [42]. Additionally, correlation coefficient values closer to 1 indicate a stronger similarity between the two rankings. The Spearman-rank and Kendall-rank are both correlation coefficients that depend only on the ranks of the variables (e.g., the nodes), and not on the observed values (e.g., the centrality values). Spearman’s correlation coefficient (*ρ*) is equivalent to the traditional linear correlation coefficient calculated on the rankings of variables, while Kendall’s correlation coefficient (*τ*) is proportional to the number of pairwise adjacent inversions that are required to convert one ranking into the other [43]. Although Kendall’s *τ* differs in magnitude and is usually smaller when compared to Spearman’s *ρ*, it has become a standard statistic to compare the correlation between two rankings for a number of reasons, such as its fast-computational time [44]. In this paper, both rank correlation coefficients are used to evaluate the correctness of the rankings to better assess the effectives of the centrality measures.

### 4.1 Spearman-rank correlation coefficient

The Spearman-rank correlation coefficient *ρ* [45] of the rankings with respect to any two centrality measures, say *C*_*X*_ and *C*_*Y*_, is calculated as follows. First, the ranking of nodes in decreasing order of the centrality values is obtained. The index at which a node appears in the list is initially considered the tentative ranking of the node. If two or more nodes have the same centrality value, the tie between the nodes is broken in favor of the node with the smaller numerical node identification label. For example, if nodes 4 and 6 both had the same centrality value, then node 4 is ranked ahead of node 6 due to its identification label of 4 being less than 6. The final ranking for a node with respect to a centrality measure is the same as the tentative ranking for the node only if it does not have a tie with any other node for the centrality measure. If two or more nodes have a tie with respect to a centrality measure, their final ranking is the average of the tentative rankings for the nodes with respect to the measure [46]. Define *d*_*i*_ as the difference in the final ranking for node *v*_*i*_ with respect to the two centrality measures *C*_*X*_ and *C*_*Y*_, where 1≤*i*≤*N* and *N* is the number of nodes in the network. Then the Spearman-rank correlation coefficient of the rankings with respect to two centrality measures, *C*_*X*_ and *C*_*Y*_, is calculated as $\rho (C_{X},C_{Y})=1-\frac{6\sum_{i=1}^{N}{d_{i}}^{2}}{N(N^{2}-1)}$.

### 4.2 Kendall-rank correlation coefficient

For any two centrality measures, *C*_*X*_ and *C*_*Y*_, define a pair of nodes *v*_*i*_ and *v*_*j*_ as concordant with respect to *C*_*X*_ and *C*_*Y*_ if the nodes are arranged in the same order by the measures, and discordant with respect to *C*_*X*_ and *C*_*Y*_ if the nodes are arranged in opposite order [46]. For example, if node 4 is ranked above node 6 by both measures *C*_*X*_ and *C*_*Y*_, then the node pair is concordant. Yet, if node 4 is ranked above node 6 by measure *C*_*X*_, but ranked below node 6 by measure *C*_*Y*_, then the node pair is discordant. If a pair of nodes are assigned the same ranking with respect to *C*_*X*_ and *C*_*Y*_, then the node pair is considered neither concordant nor discordant. Then the Kendall-rank correlation coefficient *τ* [47] of the rankings with respect to two centrality measures, *C*_*X*_ and *C*_*Y*_, is calculated as, $\tau (C_{X},C_{Y})=\frac{N_{C}-N_{D}}{N_{C}+N_{D}}$, where *N*_*C*_ is the total number of concordant pairs and *N*_*D*_ is the total number of discordant pairs.

### 4.3 Example calculation

To quantify the extent of the similarity in the ranking of the nodes with respect to the betweenness and heatmap centrality measures on the example network *G* in Fig 2, the Spearman-rank correlation is $\rho (C_{B},C_{HM})=1-\frac{\left(6)(30\right)}{15(225-1)}=0.946$ where *N* = 15 and $\sum_{i=1}^{15}{d_{i}}^{2}=30$, while the Kendall-rank correlation is $\tau (C_{B},C_{HM})=\frac{96-9}{96+9}=0.829$ where *N*_*C*_ = 96 and *N*_*D*_ = 9. The details of the calculation of the Spearman-rank correlation are provided in Table 2, while those for the Kendall-rank correlation are provided in Table 3.

**Table 2:** The Spearman-rank correlation for the betweenness (*C*_*B*_) and heatmap (*C*_*HM*_) centrality measures is calculated based upon the difference (*d*_*i*_) in the final ranking with respect to the rankings of *C*_*B*_ and *C*_*HM*_ for each node (*v*_*i*_) in network *G*.

Node *v*_*i*_	*C*_*B*_ Rank	*C*_*HM*_ Rank	*d*_*i*_	*d*_*i*_^2^
1	8	10	2	4
2	9	11	2	4
3	4	3	1	1
4	10	9	1	1
5	7	6	1	1
6	1	1	0	0
7	11	8	3	9
8	2	4	2	4
9	6	5	1	1
10	3	2	1	1
11	12	12	0	0
12	5	7	2	4
13	13	13	0	0
14	14	14	0	0
15	15	15	0	0
				$\sum_{i=1}^{15}{d_{i}}^{2}=30$

**Table 3:** The Kendall-rank correlation for the betweenness (*C*_*B*_) and heatmap (*C*_*HM*_) centrality measures is calculated based upon both the total number of concordant (*N*_*C*_) and discordant (*N*_*D*_) pairs observed in network *G*.

**Node *v***_***i***_	*C*_*B*_ **Rank**	*C*_*HM*_ **Rank**	*N*_*C*_	*N*_*D*_
6	1	1	14	0
8	2	4	11	2
10	3	2	12	0
3	4	3	11	0
12	5	7	8	2
9	6	5	9	0
5	7	6	8	0
1	8	10	5	2
2	9	11	4	2
4	10	9	4	1
7	11	8	4	0
11	12	12	3	0
13	13	13	2	0
14	14	14	1	0
15	15	15	0	0
**Total**			**96**	**9**

To assist in the calculation of the concordant and discordant pairs required for the Kendall-rank correlation used in the example, the nodes *v*_*i*_ in the first column of Table 3 are sorted according to their *C*_*B*_ ranking in the second column. The corresponding *C*_*HM*_ ranking of each node is listed in the third column. For example, node 8 is ranked second with respect to *C*_*B*_, but ranked fourth with respect to *C*_*HM*_. Since the nodes are already sorted with respect to their *C*_*B*_ ranking, then the number of concordant pairs *N*_*C*_ for node *v*_*i*_ equals the total number of larger ranks that exist below its *C*_*HM*_ ranking. Similarly, the number of discordant pairs *N*_*D*_ for node *v*_*i*_ equals the total number of smaller ranks that exist below its *C*_*HM*_ ranking. For example, consider node 12 which has *C*_*B*_ and *C*_*HM*_ rankings of 5 and 7, respectively. There are 8 concordant pairs because nodes 1, 2, 4, 7, 11, 13, 14, and 15 have both a *C*_*B*_ ranking below 5 and a *C*_*HM*_ ranking below 7. In addition, there are 2 discordant pairs since only nodes 5 and 9 have both a *C*_*B*_ ranking below 5 and a *C*_*HM*_ ranking above 7. Similar calculations are performed to determine the number of concordant and discordant pairs for the remaining nodes in the network, with the results displayed in the fourth and fifth columns of Table 3.

## 5 The SI model

Since the diffusion of information can be likened to the propagation of a disease, an epidemic model is proposed to track the information spreading process and identify potential super-spreaders within the network. In particular, a susceptible-infected (SI) model is utilized to simulate the spreading process and examine the spreading capability (i.e., the spreading efficiency) of the nodes within the network. In theory, the SI model identifies influential nodes based upon the idea that an influential node is more likely to have a role in passing along a disease (or analogously, information), and thus, have a stronger spreading capability [48]. The SI model has been used as a baseline model to compare the rankings of centrality measures [35–38], where the average infection efficiency of nodes is used as a measure to evaluate the effectiveness of a centrality measure [49].

In the SI model, each node belongs to one of two possible states: susceptible or infected. The susceptible state *S*(*t*) represents the number of nodes susceptible to, but not yet infected by, the disease at time step *t*. The infected state *I*(*t*) represents the number of nodes that have been infected and are able to spread the disease to susceptible nodes at time step *t*. Infected nodes can infect susceptible nodes with a fixed probability, and once a node becomes infected, it remains infected. Initially, all nodes are in the susceptible state, with the exception of one node in the infected state. At each time step *t*, for each infected node, one randomly selected susceptible neighbor becomes infected with probability *β*. In this paper, the value of the infection probability is set to *β* = 1 for simplicity in the subsequent experiments. The cumulative number of infected nodes at time step *t*, denoted by *F*(*t*), is used as a measure of the initially infected node’s influence at time *t*. As the time step *t* increases, *F*(*t*) increases and eventually stabilizes at time step *t*_*c*_, denoted by *F*(*t*_*c*_). Therefore, the spreading capability *F*(*t*_*c*_) is used to illustrate the influence of a particular node, where a larger *F*(*t*_*c*_) indicates a stronger influence.

Although the top-10 nodes of a network may be equivalent among two measures, the ordering of the nodes may still differ. In order to demonstrate the effectiveness of the ranking with respect to one centrality measure over that with respect to another centrality, the spreading capability of the top-10 ranked nodes among each centrality measure is compared. Following a similar direction taken by Qiao et al. [35], the top-10 nodes, collectively, serve as the source of the infection. Although *F*(*t*) stabilizes at different values of *t*_*c*_ for each real-world network, the average infection capability of the top-10 nodes is calculated at each time step and used to examine the infection ability of the top-10 nodes within each centrality ranking. Because only one randomly selected susceptible neighbor becomes infected with probability *β* = 1 at each time step, the spreading process is repeated 100 times independently to eliminate any environmental randomness. It is noted that this SI model with *β* = 1 is a modified version of the standard SI model, in which all the susceptible neighbors of an infected node have a possibility to become infected [7].

## 6 Simulation and analysis

The codes and the calculation of the heatmap and well-known centrality measures, along with the spreading capability from the SI model, are generated and executed using version 1.2.4.2 of the **igraph** package in R [50]. The datasets for the real-world scale-free networks are downloaded from version 0.1.3 of the **networkdata** package [51] and version 3.0.16 of the **tnet** package [52] in R. Finally, the experiments are run in parallel and performed on the RStudio Virtual Machine provided by Pomona College. There are 2 CPUs (Intel Xeon Processor E5 v4 at 2.20 GHz) on the physical server that the RStudio Virtual Machine resides on, and each CPU has 24 cores. With 2GB provisioned per core, the RStudio server has 96 GB of RAM.

### 6.1 Datasets

#### 6.1.1 Simulated scale-free networks

To validate the computational efficiency of the proposed centrality measure, the heatmap centrality is applied to simulated Barabási-Albert (BA) scale-free networks of various size *N* and density *d*, where *d* is defined as the ratio of the number of edges present in the network to the number of possible edges in the network. The BA model is an algorithm for generating scale-free networks through the preferential attachment mechanism, in which the more connected a node is, the more likely it is to acquire new edges [53]. In particular, the network begins with *m*_0_ (in this paper, *m*_0_ = 1) nodes. With a linear preferential attachment, then at each time step, one new node is added to the network with *m*≤*m*_0_ edges that connect it to the existing nodes within the network. After *t* time steps, the BA algorithm generates a network with |*V*| = *N* = *t*+*m*_0_ nodes and |*E*| = *mt* edges.

For an undirected network with |*V*| = *N* nodes and |*E*| = *M* edges, the density *d* is defined as $d=\frac{2M}{N(N-1)}$. With *m*_0_ = 1, such that *t* = *N*−1 and *M* = *m*(*N*−1), it can be shown that the density in a BA scale-free network is $d\approx \frac{2m}{N}$ [54]. As the betweenness centrality is the slowest among the four well-known measures (refer to Section 2 for the overview of its time complexity), the betweenness measure is used to gauge whether the proposed measure can be computed in an acceptable amount of time. In order to benchmark the runtimes of both the betweenness and heatmap centralities as both the size *N* and density *d* of the network increase, BA scale-free networks of size *N* and density *d* are constructed using the function *sample_pa* from the **igraph** package by specifying both the size of the network *N* and the number of edges to add in each time step, *m*, such that $m\approx \frac{dN}{2}$. Table 4 contains the values of *m* used to simulate the scale-free networks of various size and density in the experiments. For each specified size and density, 100 scale-free networks are simulated. For each network in the set of 100 simulated networks, the runtime to execute each centrality measure on the entire network is calculated. For each set of 100 simulated networks, the mean and standard deviation of the runtimes are calculated for each measure.

**Table 4:** The value of $m\approx \frac{dN}{2}$ represents the number of edges added to each new node in each time step such that the network of size *N* is of the desired density *d*. In the experiment, a total of 30 scale-free networks of various size and density are simulated.

$m\approx \frac{dN}{2}$	*d* = 0.002	*d* = 0.004	*d* = 0.006	*d* = 0.008	*d* = 0.01	*d* = 0.02
***N* = 1000**	1	2	3	4	5	10
***N* = 2000**	2	4	6	8	10	20
***N* = 3000**	3	6	9	12	15	30
***N* = 4000**	4	8	12	16	20	40
***N* = 5000**	5	10	15	20	25	50

For each set of 100 simulated networks, the mean and standard deviation of the basic structural features of networks of size *N* and density *d* are calculated and are summarized in Tables 5–7, where <*C*_*D*_> denotes the average degree of a node, <*cc*> denotes the clustering coefficient (i.e., a measure of the degree to which nodes in a network tend to cluster together), and diameter denotes the longest path of the shortest path between any two nodes.

**Table 5:** The mean and standard deviation of the average degree <*C*_*D*_> for each network size and density calculated from the 100 simulated networks.

<*C*_*D*_>	*d* = 0.002	*d* = 0.004	*d* = 0.006	*d* = 0.008	*d* = 0.01	*d* = 0.02
***N* = 1000**	1.998±0	3.994±0	5.988±0	7.98±0	9.97±0	19.89±0
***N* = 2000**	3.997±0	7.99±0	11.979±0	15.964±0	19.945±0	39.79±0
***N* = 3000**	5.996±0	11.986±0	17.97±0	23.948±0	29.92±0	59.69±0
***N* = 4000**	7.995±0	15.982±0	23.961±0	31.932±0	39.895±0	79.59±0
***N* = 5000**	9.994±0	19.978±0	29.952±0	39.916±0	49.87±0	99.49±0

**Table 6:** The mean and standard deviation of the clustering coefficient <*cc*> for each network size and density calculated from the 100 simulated networks.

<*cc*>	*d* = 0.002	*d* = 0.004	*d* = 0.006	*d* = 0.008	*d* = 0.01	*d* = 0.02
***N* = 1000**	0±0	0.010±0.001	0.018±0.001	0.025±0.001	0.032±0.001	0.059±0.001
***N* = 2000**	0.006±0.001	0.014±0.001	0.022±0.001	0.029±0.001	0.035±0.001	0.062±0
***N* = 3000**	0.007±0	0.016±0	0.023±0	0.030±0	0.036±0	0.063±0
***N* = 4000**	0.008±0	0.017±0	0.024±0	0.031±0	0.037±0	0.064±0
***N* = 5000**	0.009±0	0.017±0	0.025±0	0.031±0	0.037±0	0.064±0

**Table 7:** The mean and standard deviation of the diameter for each network size and density calculated from the 100 simulated networks.

**Diameter**	*d* = 0.002	*d* = 0.004	*d* = 0.006	*d* = 0.008	*d* = 0.01	*d* = 0.02
***N* = 1000**	20.12±1.908	7.84±0.368	6±0	5.02±0.141	5±0	4±0
***N* = 2000**	8.05±0.219	5.87±0.338	5±0	4.03±0.171	4±0	3±0
***N* = 3000**	6.98±0.141	5±0	4.03±0.171	4±0	4±0	3±0
***N* = 4000**	6±0	5±0	4±0	4±0	4±0	3±0
***N* = 5000**	5.46±0.501	4.04±0.197	4±0	4±0	3.42±0.496	3±0

### 6.2 Real-world scale-free networks

To validate its effectiveness in identifying influential nodes, the heatmap centrality is applied to four real-world scale-free networks: (1) Email, the email communication network of a university in Spain [55], (2) Polblogs, the hyperlink network about the political blogs in the United States [56], (3) USFlights, the airline network consisting of the airports in the United States in 2010 [57], and (4) Facebook, the social network containing the online interactions between students at University of California, Irvine [58].

Email is an undirected and unweighted network that depicts the email communication network at the University of Rovira i Virgili in Tarragona in the south of Catalonia in Spain. In the network, each node represents a user and each edge represents that at least one email was sent between two connected users.

Polblogs is an undirected and unweighted network that depicts the United States political blogosphere data compiled by Lady Adamic and Natalie Glance. In the network, each node represents a blog and each edge represents a hyperlink between two blogs. To create the Polblogs network, the largest connected component from the original network was selected, which lowered the number of nodes by 268 from 1490 to 1222. All 268 nodes that were removed were isolated in the original network. In addition, all edges, which were originally directed, were made undirected. Finally, the network was made simple by removing all multi-edges and loops, which lowered the number of edges by 3 from 16717 to 16714.

USFlights is an undirected and unweighted network of the United States’ airports in 2010, where each node represents an airport and an edge is a direct route between two airports. To create the USFlights network, the largest connected component from the original network was selected, which lowered the number of nodes by 286 from 1858 to 1572. All 286 nodes that were removed were isolated in the original network. In addition, all edges, which were originally directed, were made undirected. Finally, the network was made simple by removing all multi-edges and loops, which lowered the number of edges by 11020 from 28234 to 17214.

Facebook is an undirected and unweighted network of the online social interactions that originated from a virtual community among students at UC Irvine. Each node represents a user and an edge represents that at least one message was sent between two users. To create the Facebook network, the largest connected component from the original network was selected, which lowered the number of nodes by 6 from 1899 to 1893. All 6 nodes that were removed were isolated in the original network. In addition, all edges, which were originally directed, were made undirected. Finally, the network was made simple by removing all multi-edges and loops, which lowered the number of edges by 47899 from 61724 to 13835.

**Fig 3:**
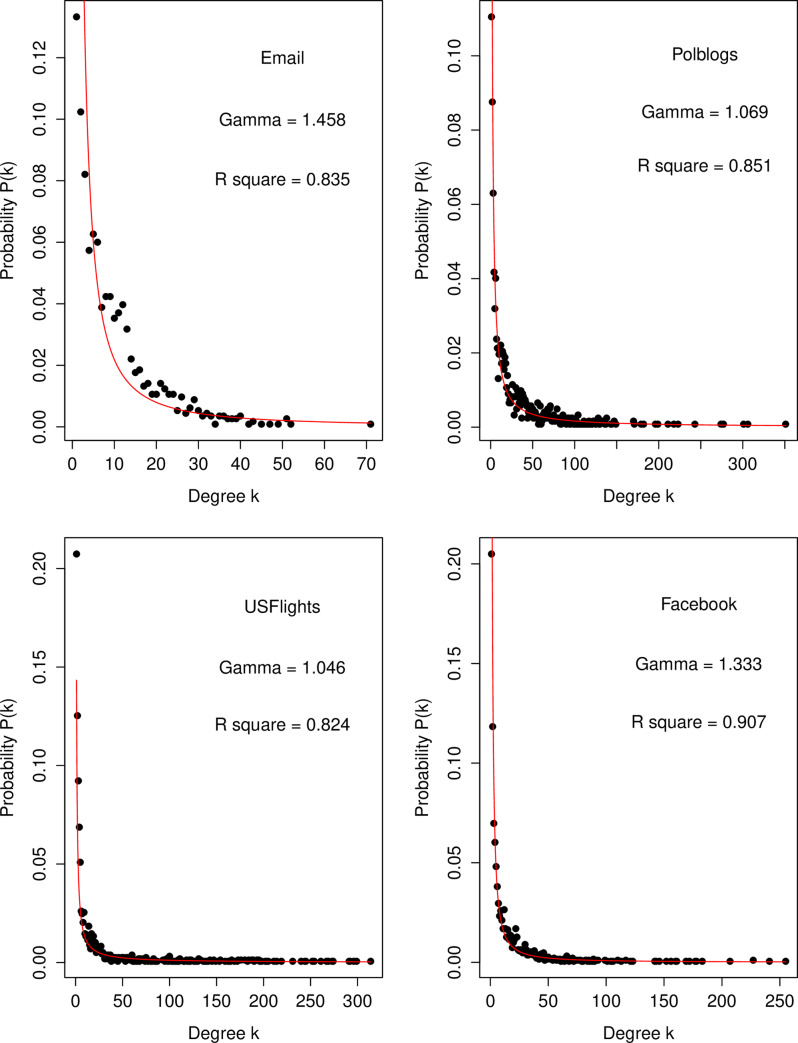
The degree distribution of the four real-world networks. The degree distribution of each real-world network exhibits a power law distribution *P*(*k*)~*k*^−*γ*^, where *γ*>1. The *R*^2^ calculated from the linear regression analysis on log(*P*(*k*))~ −*γ*log(*k*) is provided for each network as a measure of the goodness of fit for the power law model on the degree distribution. The closer *R*^2^ is to 1, the higher the degree of fit to the power-law distribution.

Fig 3 displays the degree distribution of each network, where each distribution exhibits a power law with *P*(*k*)~*k*^−*γ*^, where *γ*>1, such that each network can be conjectured to be scale-free. The basic structural features of the four real-world networks are summarized in Table 8, where *N* and *M* denote the number of nodes and edges, respectively, in the network, *d* denotes the density, <*C*_*D*_> denotes the average degree of a node, <*cc*> denotes the clustering coefficient, and diameter denotes the longest path of the shortest path between any two nodes.

**Table 8:** The basic structural features of the four real-world scale-free networks.

Dataset	Network Type	*N*	*M*	*d*	<*C*_*D*_>	<*cc*>	Diameter
Email	Communication	1133	5451	0.0085	9.622	0.166	8
Polblogs	Hyperlink	1222	16714	0.0224	27.355	0.226	8
USFlights	Airline	1572	17214	0.0139	21.901	0.384	8
Facebook	Social	1893	13835	0.0077	14.617	0.057	8

### 6.3 Efficiency analysis on simulated scale-free networks

The efficiency of the heatmap centrality is demonstrated by comparing its CPU (central processing unit) time for the simulated scale-free networks described in Tables 4–7 against the runtime of the betweenness centrality. In addition, the effectiveness of the heatmap centrality is detailed by comparing both the Spearman-rank and Kendall-rank correlations of its ranking with respect to the rankings of the degree, eigenvector, closeness, and betweenness centralities on the same set of simulated scale-free networks.

### 6.4 Experiment 1: compare the CPU time

In this experiment, the function *proc*.*time* in version 3.6.0 of the **base** package in R [59] is used to measure the CPU time (in seconds) required to execute the betweenness and heatmap centrality measures on each simulated scale-free network of size *N* and density *d*. The CPU time is defined as the sum of the “user time” and “system time” values, where “user time” is the CPU time charged from the execution of user instructions of the calling process [59], and “system time” is the CPU time charged for execution by the system on behalf of the calling process [59]. The “user + system” value indicates how much CPU has been used to execute the algorithm and calculate the centrality value of each of the *N* nodes in the simulated scale-free network. The betweenness centrality is calculated using the function *betweenness* in the **igraph** package [50]. The pseudocode for the heatmap centrality is provided in Fig 1. The functions *closeness* and *degree* in the **igraph** package are used to calculate the closeness and degree centrality, respectively, that are required in the calculation of the heatmap centrality.

**Fig 4:**
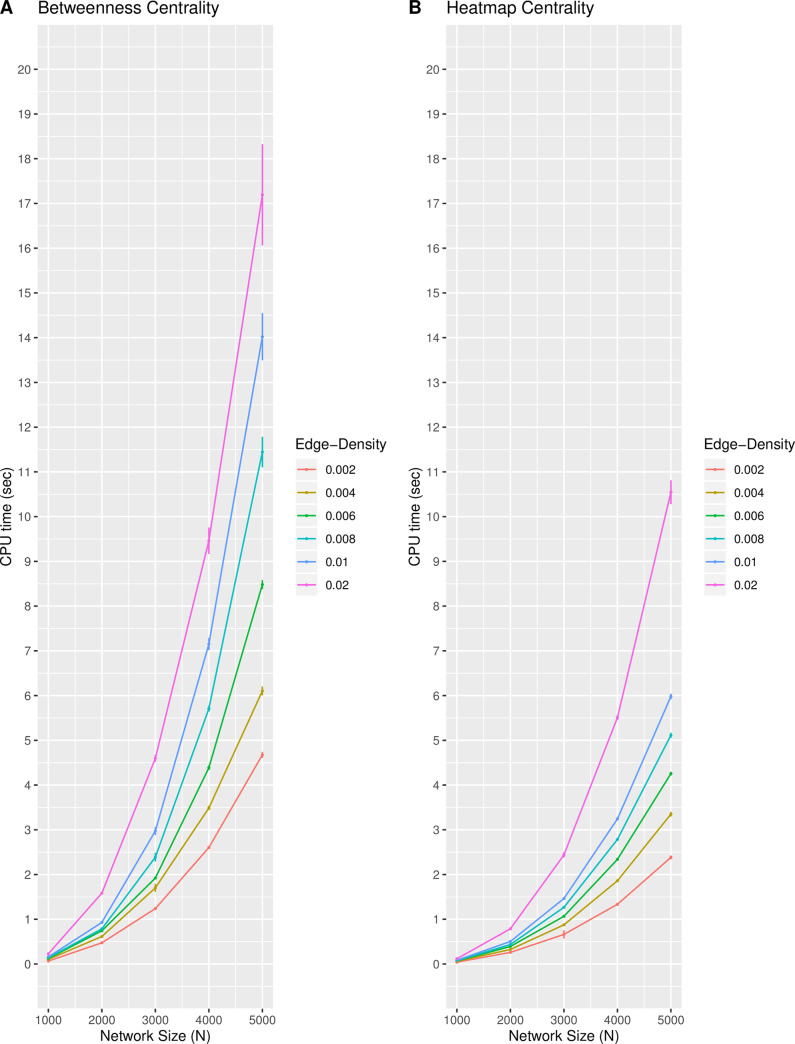
The CPU time for the centrality measures. The CPU time (in seconds) of the both the (A) betweenness and (B) heatmap centrality measures required to calculate the value of each node in the scale-free networks of size *N* and density *d* averaged over 100 iterations. The standard deviation of the CPU times at each point is included.

Fig 4 displays the mean CPU time for each of the two centrality measures on the scale-free networks of size *N* and density *d*, averaged from 100 iterations. The standard deviation of the CPU time for each measure per network size and density from the 100 iterations is included in Fig 4 as error bars to represent the variability. As both the size and density of the scale-free network increases in Fig 4, the heatmap centrality requires nearly half the CPU time than the betweenness centrality. Although the heatmap and betweenness centralities share the same time complexity, it is noted that the magnitude of the observed differences in CPU times may vary with different function implementations and versions of the packages in R.

### 6.5 Experiment 2: compare the correlation of the rankings

**Fig 5:**
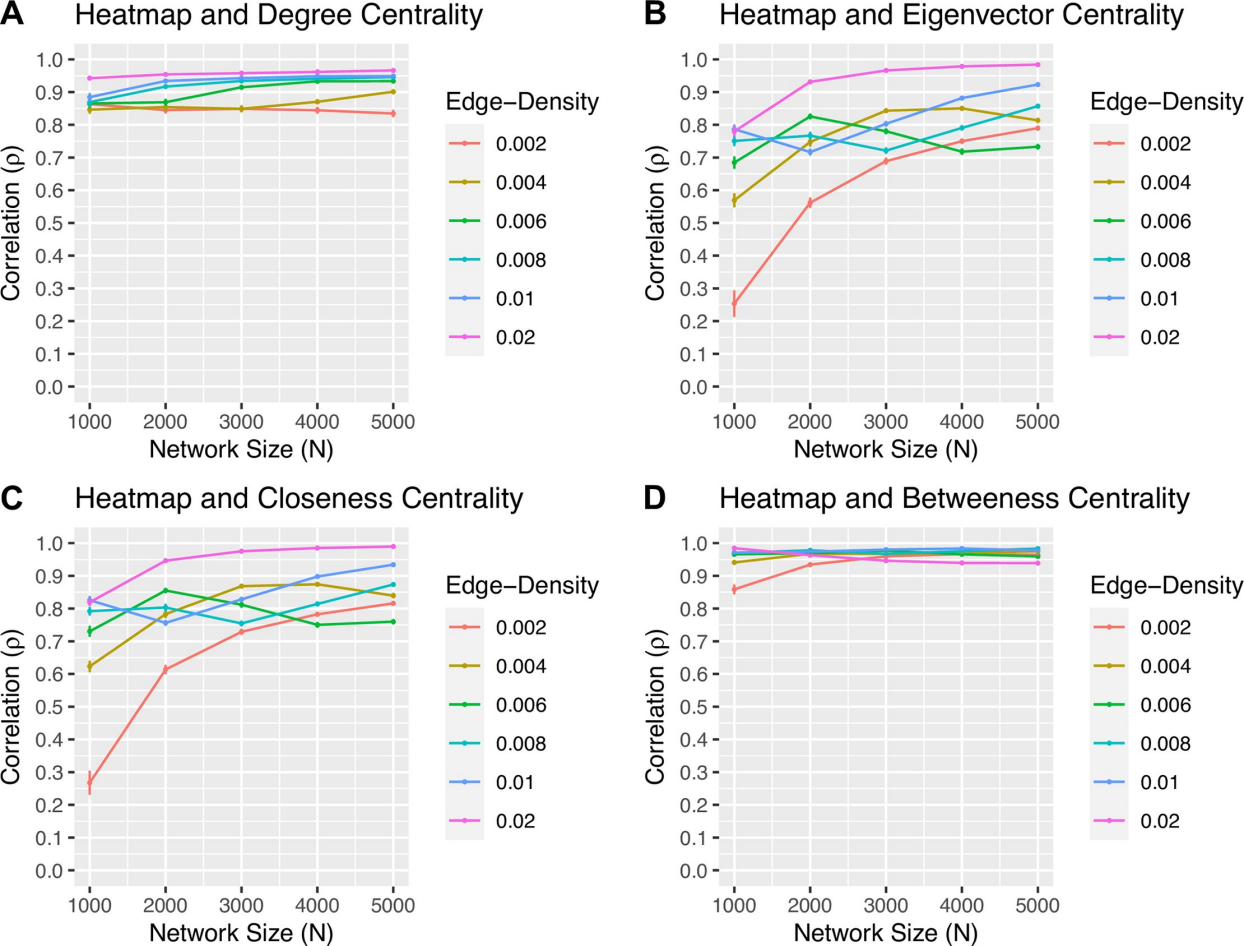
The Spearman-rank correlation coefficient *ρ*. The value of *ρ* for the rankings with respect to the (A) heatmap and degree, (B) heatmap and eigenvector, (C) heatmap and closeness, and (D) heatmap and betweenness centrality measures applied to each simulated scale-free network of size *N* and density *d*. The standard deviation of *ρ* at each point is included.

**Fig 6:**
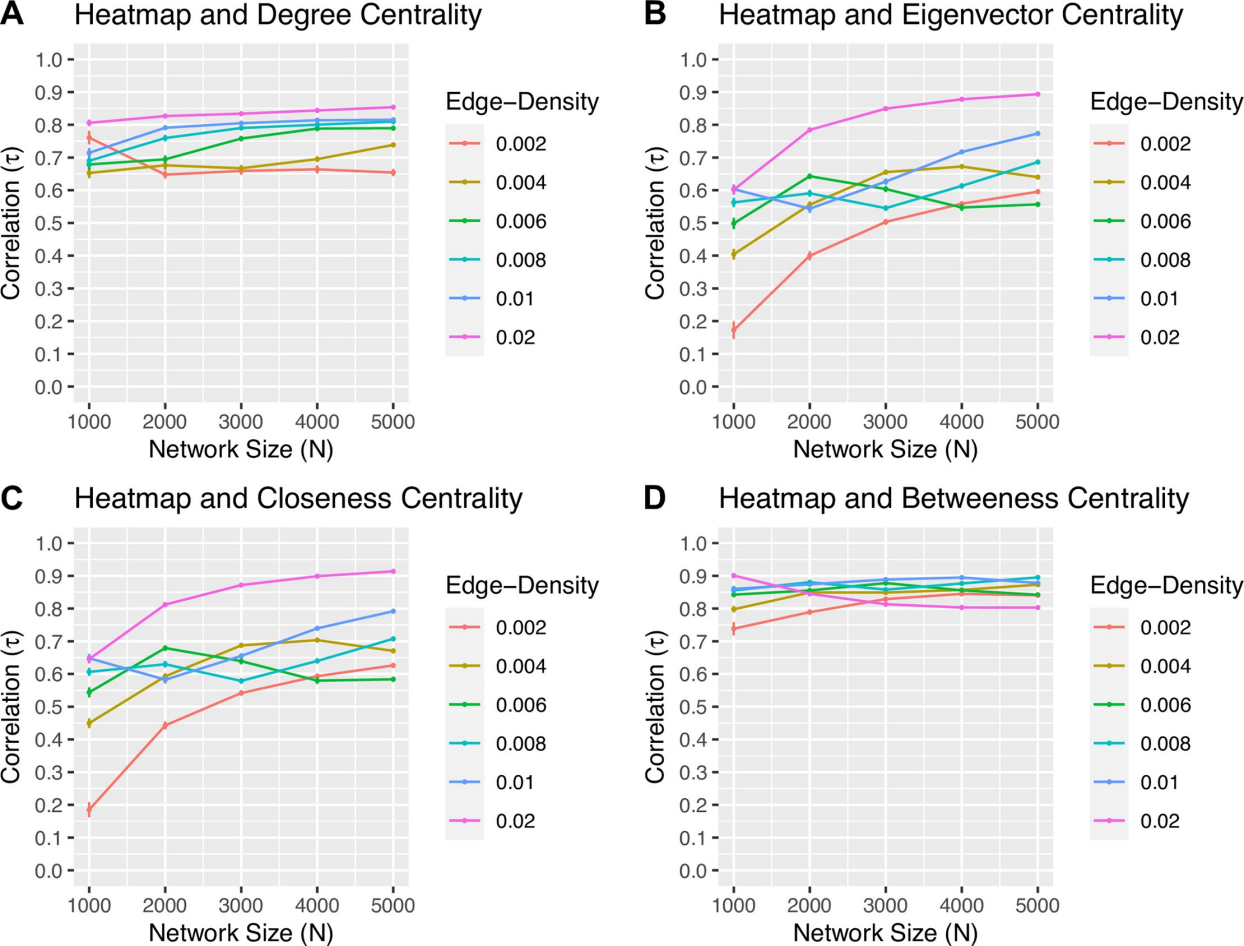
The Kendall-rank correlation coefficient *τ*. The value of *τ* for the rankings with respect to the (A) heatmap and degree, (B) heatmap and eigenvector, (C) heatmap and closeness, and (D) heatmap and betweenness centrality measures applied to each simulated scale-free network of size *N* and density *d*. The standard deviation of *τ* at each point is included.

For each simulated scale-free network of size *N* and density *d*, the Spearman-rank and Kendall-rank correlations of the heatmap centrality with respect to the degree, eigenvector, closeness, and betweenness measures are calculated. The results of the Spearman-rank correlation are shown in Fig 5, while those of the Kendall-rank are shown in Fig 6. In this experiment, both rank correlation coefficients highlight similar relationships between each pair of centrality measures, although the values of Kendall’s *τ* tend to be smaller than that of Spearman’s *ρ*. In particular, the Spearman-rank correlation between the rankings of the heatmap and degree centralities in Fig 5 is strong. With the exception of a few network sizes and densities, as both the size and density increase, the value of *ρ* for both the heatmap and closeness centralities, and the heatmap and eigenvector centralities increases. Finally, the correlation of the rankings with respect to the betweenness and heatmap centralities is the strongest amongst all other correlations, with the value of *ρ* suggesting a very strong association between the rankings of the two measures regardless of the value of *N* or *d*. Similar conclusions can be drawn from the Kendall-rank correlation between the rankings of the centrality measures depicted in Fig 6.

### 6.6 Experimental analysis on real-world scale-free networks

To verify the efficiency of the heatmap centrality on real-world scale-free networks, several experiments are conducted to examine the relative advantages and disadvantages. In order to evaluate the efficiency of the proposed measure, four centrality measures which comprise of degree, eigenvector, closeness, and betweenness are also applied to the same set of real-world networks for comparison. The experiments include (1) identifying the ten most influential nodes within each network, (2) measuring the effectiveness of the top-10 nodes with respect to each centrality ranking using a modified susceptible-infected (SI) model, and (3) calculating the correlation of the rankings with respect to each pair of centrality measures. The real-world networks of varying sizes and densities include (1) Email, (2) Polblogs, (3) USFlights, and (4) Facebook, where the basic structural features of the four real-world networks are provided in Table 8.

### 6.7 Experiment 1: compare the top-10 ranked nodes

In this experiment, the proposed heatmap centrality measure (*C*_*HM*_), degree centrality (*C*_*D*_), eigenvector centrality (*C*_*E*_), closeness centrality (*C*_*C*_), and betweenness centrality (*C*_*B*_) are employed to identify the top-10 nodes of the four real-world networks: Email, Polblogs, USFlights, and Facebook. The results are shown in Tables 9–12. With respect to the centrality measures *C*_*D*_, *C*_*E*_, *C*_*C*_, and *C*_*B*_, the top-ranked node has the most positive value, while the top-ranked node according to the heatmap centrality *C*_*HM*_ has the most negative value. In each table, the nodes that have been identified by all five measures are bolded.

**Table 9:** The top-10 ranked nodes of the Email network with respect to the degree (*C*_*D*_), eigenvector (*C*_*E*_), closeness (*C*_*C*_), betweenness (*C*_*B*_), and heatmap (*C*_*HM*_) centrality measures. The nodes in bold are identified by all five measures.

**Email**					
**Rank**	*C*_*D*_	*C*_*E*_	*C*_*C*_	*C*_*B*_	*C*_*HM*_
1	**100**	**100**	**310**	**310**	512
2	**310**	16	23	**100**	23
3	16	183	**100**	23	**100**
4	23	191	**38**	512	219
5	**38**	**38**	37	72	72
6	37	45	72	219	**310**
7	183	52	219	127	127
8	219	109	48	37	37
9	21	**310**	127	332	**38**
10	72	3	353	**38**	504

**Table 10:** The top-10 ranked nodes of the Polblogs network with respect to the degree (*C*_*D*_), eigenvector (*C*_*E*_), closeness (*C*_*C*_), betweenness (*C*_*B*_), and heatmap (*C*_*HM*_) centrality measures. The nodes in bold are identified by all five measures.

**Polblogs**					
**Rank**	*C*_*D*_	*C*_*E*_	*C*_*C*_	*C*_*B*_	*C*_*HM*_
1	**127**	**127**	**838**	672	**127**
2	**838**	**48**	**127**	**127**	672
3	672	**497**	497	768	**497**
4	**48**	**566**	**48**	**838**	**838**
5	**497**	283	890	**497**	768
6	768	147	**566**	1178	**48**
7	1006	**838**	768	**48**	890
8	**566**	84	922	782	**566**
9	922	384	1178	922	922
10	1178	498	672	**566**	1178

**Table 11:** The top-10 ranked nodes of the USFlights network with respect to the degree (*C*_*D*_), eigenvector (*C*_*E*_), closeness (*C*_*C*_), betweenness (*C*_*B*_), and heatmap (*C*_*HM*_) centrality measures. The nodes in bold are identified by all five measures.

**USFlights**					
**Rank**	*C*_*D*_	*C*_*E*_	*C*_*C*_	*C*_*B*_	*C*_*HM*_
1	**97**	1016	**97**	76	427
2	604	**97**	754	427	76
3	1016	368	907	754	39
4	754	604	333	654	60
5	654	907	604	866	**97**
6	333	335	654	1219	682
7	422	1062	841	584	754
8	907	606	1016	333	654
9	606	422	422	**97**	1074
10	866	176	606	907	604

**Table 12:** The top-10 ranked nodes of the Facebook network with respect to the degree (*C*_*D*_), eigenvector (*C*_*E*_), closeness (*C*_*C*_), betweenness (*C*_*B*_), and heatmap (*C*_*HM*_) centrality measures. The nodes in bold are identified by all five measures.

**Facebook**					
**Rank**	*C*_*D*_	*C*_*E*_	*C*_*C*_	*C*_*B*_	*C*_*HM*_
1	**103**	**103**	**32**	**9**	**105**
2	**9**	**105**	**105**	**398**	**32**
3	**105**	**32**	**9**	**105**	**9**
4	**398**	**9**	**3**	**32**	**3**
5	**32**	247	636	**103**	**398**
6	41	636	**103**	42	521
7	**3**	**398**	42	**3**	42
8	42	**3**	247	41	636
9	247	370	596	521	711
10	636	194	**398**	247	**103**

According to the results shown in Table 9, in the Email network, the proposed measure *C*_*HM*_ shares the same seven, three, eight, and nine nodes between *C*_*D*_, *C*_*E*_, *C*_*C*_, and *C*_*B*_ respectively. Based upon the results shown in Table 10, in the Polblogs network, the number of the same nodes in the top-10 ranking between the heatmap centrality and *C*_*D*_, *C*_*E*_, *C*_*C*_, and *C*_*B*_ is nine, five, ten, and nine, respectively. From the results in Table 11, in the USFlights network, the proposed measure *C*_*HM*_ shares the same four, two, four, and five nodes between *C*_*D*_, *C*_*E*_, *C*_*C*_, and *C*_*B*_ respectively. Finally, based upon the results shown in Table 12, in the Facebook network, the number of the same nodes in the top-10 ranking between the heatmap centrality and *C*_*D*_, *C*_*E*_, *C*_*C*_, and *C*_*B*_ is eight, seven, eight, and eight, respectively.

With the exception the results taken from the USFlights network, the heatmap centrality shares a minimum of seven of the top-10 ranked nodes with the degree, closeness, and betweenness centralities, respectively. Finally, with respect to the eigenvector centrality, the heatmap centrality shares only three to seven of the top-10 ranked nodes.

### 6.8 Experiment 2: compare the average spreading capability of the top-10 ranked nodes

**Fig 7:**
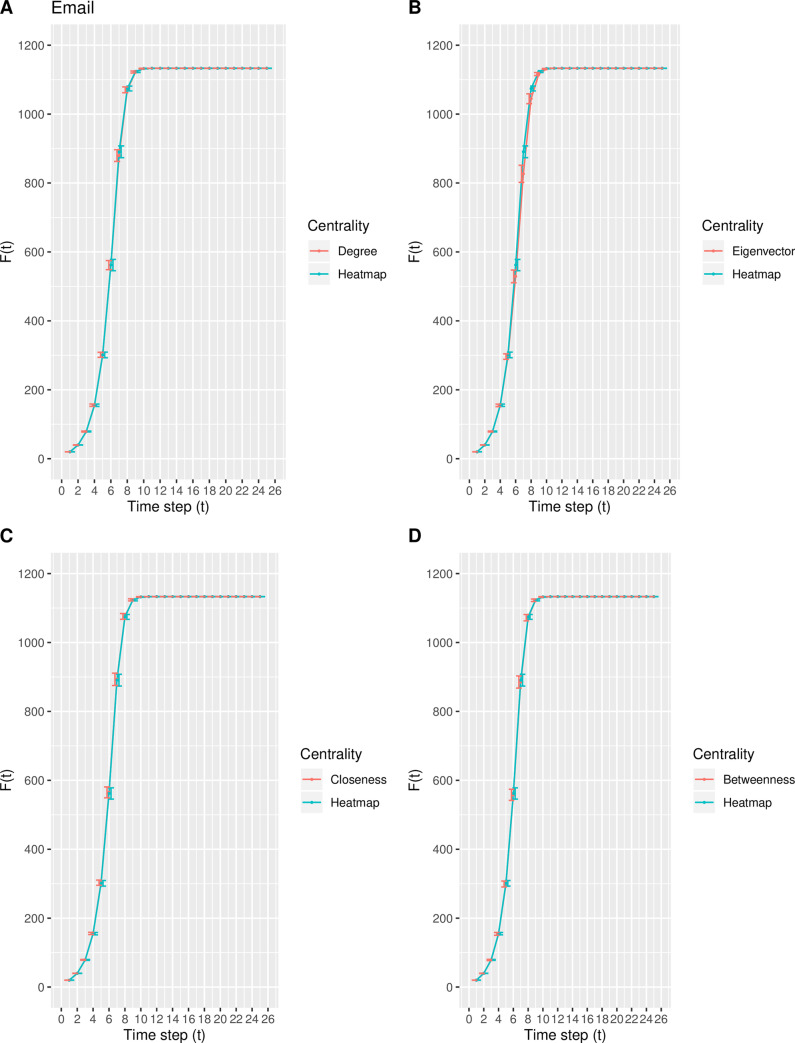
The spreading capability *F*(*t*) in the Email network. A comparison of *F*(*t*) of the top-10 nodes in the Email network between the heatmap centrality and the (A) degree, (B) eigenvector, (C) closeness, and (D) betweenness centrality measures. The standard deviation of *F*(*t*) at each point is included.

**Fig 8:**
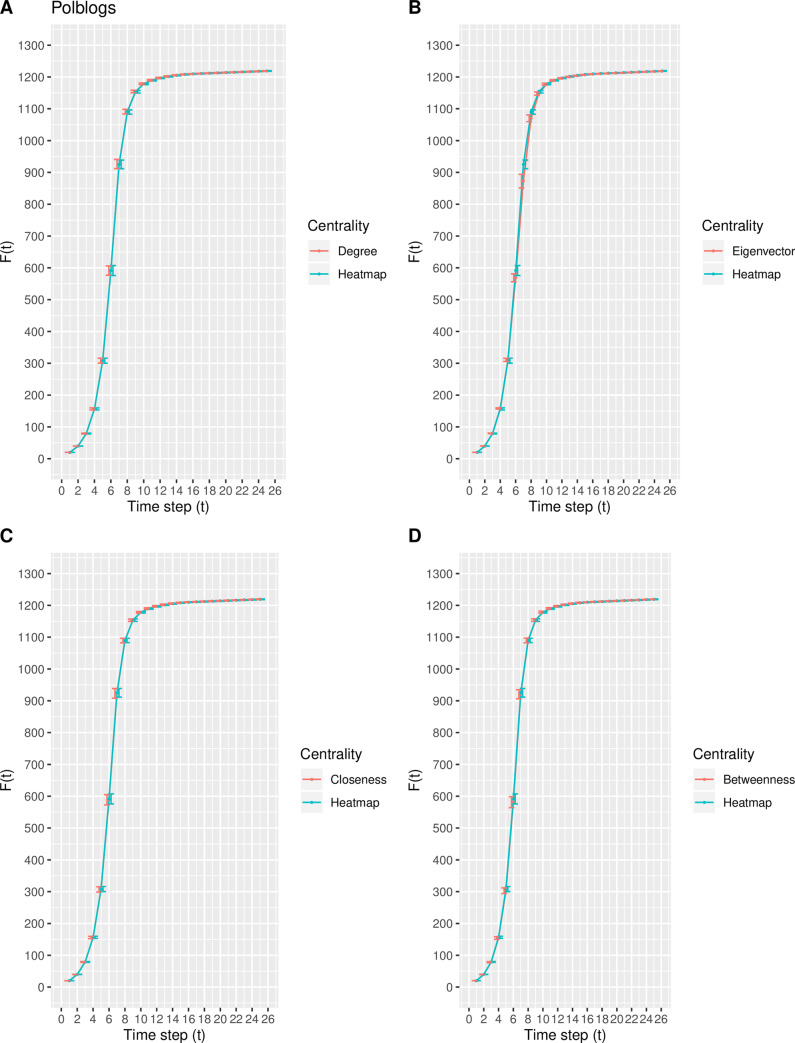
The spreading capability *F*(*t*) in the Polblogs network. A comparison of *F*(*t*) of the top-10 nodes in the Polblogs network between the heatmap centrality and the (A) degree, (B) eigenvector, (C) closeness, and (D) betweenness centrality measures. The standard deviation of *F*(*t*) at each point is included.

**Fig 9:**
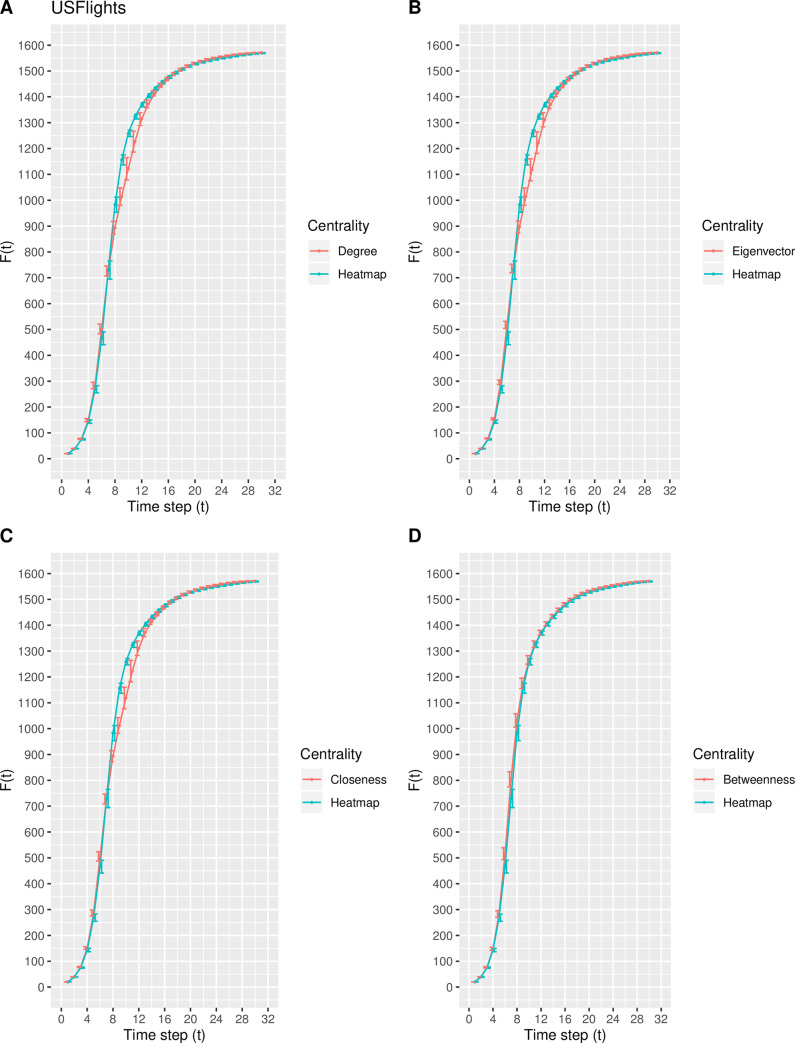
The spreading capability *F*(*t*) in the USFlights network. A comparison of *F*(*t*) of the top-10 nodes in the USFlights network between the heatmap centrality and the (A) degree, (B) eigenvector, (C) closeness, and (D) betweenness centrality measures. The standard deviation of *F*(*t*) at each point is included.

**Fig 10:**
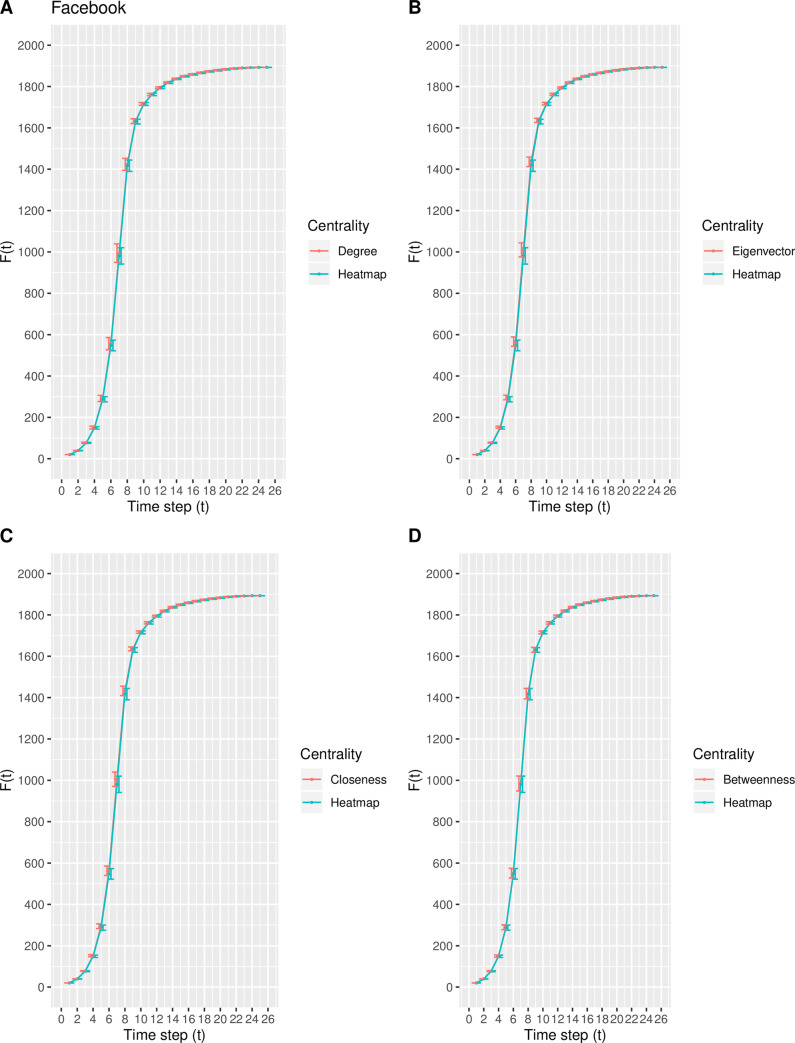
The spreading capability *F*(*t*) in the Facebook network. A comparison of *F*(*t*) of the top-10 nodes in the Facebook network between the heatmap centrality and the (A) degree, (B) eigenvector, (C) closeness, and (D) betweenness centrality measures. The standard deviation of *F*(*t*) at each point is included.

In this experiment, a modification of the susceptible-infected (SI) model is used to estimate the spreading capability (i.e., spreading influence) of the top-10 nodes ranked by the degree, eigenvector, closeness, betweenness, and heatmap centrality measures. Inspired by the direction of Qiao et al. [35], the top-10 nodes ranked by each centrality measure collectively serve as the set of initially infected nodes. As the time step *t* increases, the total number of infected nodes *F*(*t*) increases and finally stabilizes at time step *t*_*c*_, at which time there are no susceptible nodes within the network. Due to the size of each real-world network, the value of *t*_*c*_ varies such that *F*(*t*) eventually stabilizes around *t*_*c*_ = 25, 25, 30, and 25 time steps for the Email, Polblogs, USFlights, and Facebook networks, respectively. The mean and standard deviation of *F*(*t*) for each centrality measure is calculated from the 100 iterations. Figs 7–10 depict the mean spreading capability *F*(*t*) of the top-10 nodes with respect to each centrality measure for each real-world network, where the standard deviation is included as error bars to represent the variability.

From Fig 7, in the Email network, the proposed model shows similar spreading efficiency with degree, closeness, and betweenness, while outperforming that of the eigenvector centrality. In the Polblogs network, the curve generated from the proposed measure in Fig 8 is nearly identical to that from the degree, closeness, and betweenness centralities, while outperforming that of eigenvector centrality. The result from Fig 8 is not surprising, as the same nine of the top-10 nodes are identified by the degree, closeness, betweenness, and heatmap centralities. From Fig 9, in the USFlights network, the curve produced by the heatmap centrality is steeper in comparison with the curves of degree, eigenvector, and closeness, but is similar to the curve produced by the betweenness centrality. Finally, from Fig 10, a similar spreading efficiency is demonstrated among all five measures, with the curves almost all overlapping. In conclusion, the heatmap centrality presents similar spreading efficiency performances, overall, when compared with the other measures.

### 6.9 Experiment 3: compare the correlation of the rankings

To quantify the correctness of the rankings with respect to the centrality measures in real-world scale-free networks, both the Spearman-rank and Kendall-rank correlation coefficients are adopted, and the results are displayed in Tables 13 and 14, respectively. Among the correlation of the rankings with respect to the centrality measures, the values of both *ρ* and *τ* are strongest for the betweenness and heatmap centralities. In particular, the correlations among the four real-world networks are 0.88<*ρ*(*C*_*HM*_, *C*_*B*_)<0.98 and 0.70<*τ*(*C*_*HM*_, *C*_*B*_)<0.88. In a comparison with the remaining three measures, the ranking of the heatmap measure is more highly correlated with that of degree while less correlated with that of the eigenvector centrality. In summary, while displaying a strong correlation with the degree centrality, the heatmap centrality possesses the strongest correlation with the betweenness measure in each of the real-world networks.

**Table 13:** The Spearman-rank correlation *ρ* of the rankings of the heatmap (*C*_*HM*_) centrality with respect to the rankings of the degree (*C*_*D*_), eigenvector (*C*_*E*_), closeness (*C*_*C*_), and betweenness (*C*_*B*_) centrality measures, respectively, for each of the four real-world networks. For each network, the value of *ρ*(*C*_*B*_,*C*_*HM*_) is the largest.

**Network**	*ρ*(*C*_*D*_,*C*_*HM*_)	*ρ*(*C*_*E*_,*C*_*HM*_)	*ρ*(*C*_*C*_,*C*_*HM*_)	*ρ*(*C*_*B*_,*C*_*HM*_)
Email	0.934	0.789	0.858	**0.976**
Polblogs	0.938	0.873	0.925	**0.962**
USFlights	0.873	0.451	0.557	**0.883**
Facebook	0.960	0.900	0.894	**0.977**

**Table 14:** The Kendall-rank correlation *τ* of the rankings of the heatmap (*C*_*HM*_) centrality with respect to the rankings of the degree (*C*_*D*_), eigenvector (*C*_*E*_), closeness (*C*_*C*_), and betweenness (*C*_*B*_) centrality measures, respectively, for each of the four real-world networks. For each network, the value of *τ*(*C*_*B*_,*C*_*HM*_) is the largest.

**Network**	*τ*(*C*_*D*_,*C*_*HM*_)	*τ*(*C*_*E*_,*C*_*HM*_)	*τ*(*C*_*C*_,*C*_*HM*_)	*τ*(*C*_*B*_,*C*_*HM*_)
Email	0.795	0.594	0.675	**0.870**
Polblogs	0.805	0.694	0.783	**0.834**
USFlights	0.696	0.351	0.404	**0.702**
Facebook	0.850	0.740	0.731	**0.876**

## 7 Discussion

Motivated by a different interpretation of the “shortest path” between two nodes, this paper aims to explore the properties of a new centrality measure, the heatmap centrality, as a potentially viable measure in the identification of super-spreader nodes in scale-free networks. Although high degree nodes, high betweenness nodes, and high closeness nodes have been identified as good initial spreaders, the heatmap centrality utilizes features from all three measures to strike a balance between accuracy and algorithmic simplicity in the identification of the super-spreader nodes within real-world networks. By definition, the heatmap centrality may be considered a “shortest path” based measure, in that it identifies an influential node as one with a higher likelihood of having information pass through the particular node by considering the difference in the node’s farness and the average sum of the farness of its adjacent neighbors. To verify the effectiveness of the heatmap centrality, two experiments based upon simulated scale-free networks and three experiments based upon four real-world scale-free networks are conducted.

The results of the experiments based upon the simulated scale-free networks are:

1. The proposed heatmap centrality on sparse networks can be computed in an acceptable amount of time as both the size and density of the network increase.
2. With the exception of a few network sizes and densities, the heatmap measure exhibits an increasing correlation with respect to the eigenvector and closeness centralities as both the size and density of the network increase.
3. Regardless of the size or density of the network, the correlations among the heatmap with respect to the degree and betweenness centralities are strong.
4. The correlation among the heatmap and betweenness centrality is the highest correlation among any other pair of measures.

The results of the experiments based upon the real-world scale-free networks are:

1. The heatmap centrality shares, at minimum, seven of the top-10 ranked nodes identified by the degree, closeness, and betweenness centralities, respectively.
2. Using a modification of the standard SI model, the heatmap centrality presents similar spreading efficiency performances when compared with the spreading efficiency of the other measures.
3. The degree and betweenness centrality measures each possess a strong correlation with the heatmap centrality.
4. In comparison to the other measures, the heatmap centrality is most strongly correlated with the betweenness centrality.

In summary, the properties of the heatmap centrality as a potential measure to identify super-spreader nodes in scale-free networks are highlighted through the experimental results. In particular, the proposed measure may be executed in acceptable amount of CPU time, can successfully identify top-10 nodes, and possesses the strongest correlation with the betweenness centrality measure.

In this paper, the proposed heatmap centrality measure is applied to undirected and unweighted scale-free networks. Yet, a real-world scale-free network may be directed and/or weighted. Therefore, future research includes an evaluation of the proposed measure on direct and weighted scale-free networks, as well as on other network models, such as random networks or small-world networks. In addition, future work includes exploring the relationship of the heatmap centrality with other diffusion and flow-based centrality measures, such as random-walk betweenness, flow betweenness, random walk, entropy, and information centrality.
